# *In Vitro* and *In Vivo* Evaluation of Synergistic Effects of Everolimus in Combination with Antifungal Agents on *Exophiala dermatitidis*

**DOI:** 10.1128/spectrum.05302-22

**Published:** 2023-05-04

**Authors:** Gengpei Jia, Jing Hu, Lihua Tan, Longting Li, Lujuan Gao, Yi Sun

**Affiliations:** a Department of General Medicine, Jingzhou Hospital Affiliated to Yangtze University, Jingzhou, Hubei Province, China; b Department of Dermatology, Jingzhou Hospital Affiliated to Yangtze University, Jingzhou, Hubei Province, China; c Department of Reproductive Medicine, Jingzhou Hospital Affiliated to Yangtze University, Jingzhou, Hubei Province, China; d Department of Dermatology, Zhongshan Hospital (Xiamen), Fudan University, Xiamen, Fujian Province, China; e Department of Dermatology, Zhongshan Hospital, Fudan University, Shanghai, China; f Xiamen Clinical Research Center for Cancer Therapy, Xiamen, Fujian Province, China; Mayo Foundation for Medical Education and Research

**Keywords:** *E. dermatitidis*, everolimus, combination, antifungal agents, ROS, efflux pumps

## Abstract

To investigate the combined function of the novel oral mTOR inhibitor, everolimus, with antifungal agents and their potential mechanisms against *Exophiala dermatitidis*, the CLSI microliquid-based dilution method M38-A2, chequerboard technique, and disk diffusion testing were performed. The efficacy of everolimus was evaluated in combination with itraconazole, voriconazole, posaconazole, and amphotericin B against 16 clinically isolated strains of *E. dermatitidis*. The synergistic effect was determined by measuring the MIC and fractional inhibitory concentration index. Dihydrorhodamine 123 was used for the quantification of ROS levels. The differences in the expression of antifungal susceptibility-associated genes were analyzed following different types of treatment. Galleria mellonella was used as the *in vivo* model. While everolimus alone showed minimal antifungal effects, combinations with itraconazole, voriconazole, posaconazole, or amphotericin B resulted in synergy in 13/16 (81.25%), 2/16 (12.5%), 14/16 (87.75%), and 5/16 (31.25%) of isolates, respectively. The disk diffusion assay revealed that the combination of everolimus and antifungal drugs showed no significant increase in the inhibition zones compared with the single agent, but no antagonistic effects were observed. Combination of everolimus and antifungal agents resulted in increased ROS activity (everolimus + posaconazole versus posaconazole [*P* < 0.05], everolimus + amphotericin B versus amphotericin B [*P* < 0.002]). Simultaneously, compared to mono-treatment, the combination of everolimus + itraconazole suppressed the expression of *MDR2* (*P* < 0.05) and the combination of everolimus + amphotericin B suppressed the expression of *MDR3* (*P* < 0.05) and *CDR1B* (*P* < 0.02). *In vivo*, combinations of everolimus and antifungal agents improved survival rates, particularly the combination of everolimus + amphotericin B (*P* < 0.05). In summary, the *in vivo* and *in vitro* experiments performed in our study suggest that the combination of everolimus with azoles or amphotericin B can have synergistic effects against *E. dermatitidis*, potentially due to the induction of ROS activity and inhibition of efflux pumps, providing a promising new approach for the treatment of *E. dermatitidis* infections.

**IMPORTANCE** Cancer patients with *E. dermatitidis* infection have high mortality if untreated. Clinically, the conventional treatment of *E. dermatitidis* is poor due to the long-term use of antifungal drugs. In this study, we have for the first time investigated the interaction and action mechanism of everolimus combined with itraconazole, voriconazole, posaconazole, and amphotericin B on *E. dermatitidis in vitro* and *in vivo*, which provided new ideas and direction for further exploring the mechanism of drug combination and clinical treatment of *E. dermatitidis.*

## INTRODUCTION

*Exophiala dermatitidis* (*E. dermatitidis*) is an opportunistic pathogen with a global infection epidemic (1). This fungus causes a variety of diseases, including skin infections, suppurative arthritis, endocarditis, catheter-related fungemia, and systemic infections, with a mortality rate of 40% (2–4). Although skin infections are the most common, reports of deeper infections have been increasing annually (5), such as fatal cerebral phaeohyphomycosis due to central nervous system involvement (6). Currently, extensive research is being conducted worldwide on *E. dermatitidis*; however, there is no effective treatment available. According to the current case report statistics, amphotericin B, itraconazole, and voriconazole have demonstrated efficacy against 44%, 50%, and 71.4% of *E. dermatitidis* infections, respectively (7). Given the shortage of potent antifungal medications in recent years, it is encouraging to note that certain drug combinations are showing promise. However, it has been observed that the combination of amphotericin B and azole is less efficient (7). Therefore, it is critical to develop additional drugs in combination with azoles in order to improve therapeutic efficacy.

The primary target of the rapamycin inhibitor, mTOR, is cell growth regulatory molecules, which it uses to control growth (8). According to previous research, the TOR pathway regulates several processes, including proliferation, translation, transcription, autophagy, ribosome biogenesis, lipid homeostasis, morphogenesis, and cell aggregation, to facilitate the yeast cell response to nutrients, which is essential for virulence and pathogenicity (8, 9). Studies have shown that the combination of the mTOR inhibitor everolimus (EVL) and azoles has a synergistic effect on *Scedosporium*, resulting in enhanced antifungal activity of azoles (10). It is plausible that EVL could increase the efficacy of antifungal agents against *E. dermatitidis*. The purpose of this study was to further confirm the ability of EVL to enhance the efficacy of itraconazole (ITC), voriconazole (VRC), and posaconazole (POS), as well as amphotericin B (AMB). Additionally, the study aimed to investigate the feasibility of using the mTOR signaling pathway in critical fungal physiological processes as a novel antifungal drug target. The ultimate goal was to provide more theoretical research support for identifying novel drug targets against *E. dermatitidis*. We examined the effects of EVL coupled with azoles and AMB in 16 wild strains of *E. dermatitidis in vitro* and *in vivo*, as well as measured ROS activity and antifungal susceptibility-related gene expression, to further investigate the combined effects and probable mechanisms of action.

## RESULTS

### *In vitro* drug sensitivity test.

The MIC ranges of EVL, ITC, VRC, POS, and AMB were 8–>16 μg/mL, 0.25 to 1 μg/mL, 0.063 to 0.25 μg/mL, 0.125 to 2 μg/mL, and 1 to 4 μg/mL, respectively. Although EVL alone did not show significant antifungal effects, it demonstrated high rates of synergy when combined with ITC, POS, and AMB, with rates of 81.25% (13/16 of isolates), 87.5% (14/16 of isolates), and 31.25% (5/16 of isolates), respectively. At the same time, EVL and VRC also had a limited synergy effect with a rate of 12.5% (2/16 of isolates). The combination of EVL with antifungal agents resulted in decreased MICs of ITC by 4 to 8 times, VRC by 1 to 4 times, POS by 2 to 32 times, and AMB by 1 to 8 times. Furthermore, no antagonistic effect was noticed (see Table 1).

**TABLE 1:** MICs and FICIs of EVL in combination with antifungal agents^*a*^

Strains	Agent alone (μg/mL)					Combinations (μg/mL)			
	EVL	ITC	VRC	POS	AMB	EVL/iTC	EVL/vRC	EVL/pOS	EVL/aMB
109140	>16	0.5	0.25	0.5	4	2/0.125(0.313,S)	0.25/0.25(1.008,I)	1/0.063(0.156,S)	1/2(0.531,I)
109145	>16	1	0.125	0.5	4	2/0.125(0.188,S)	0.25/0.125(1.008,I)	2/0.125(0.313,S)	1/2(0.531,I)
109148	>16	0.5	0.125	0.5	4	2/0.063(0.188,S)	0.25/0.125(1.008,I)	2/0.031(0.125,S)	8/1(0.5,S)
109149	>16	0.25	0.125	2	4	8/0.063(0.5,S)	0.25/0.125(1.008,I)	2/0.063(0.313,S)	0.25/4(1.008,I)
109152	>16	0.25	0.063	0.5	4	2/0.031(0.188,S)	2/0.063(1.008,I)	1/0.031(0.094,S)	0.25/2(0.508,I)
BMU00028	16	1	0.063	1	1	2/0.25(0.375,S)	0.25/0.063(1.016,I)	2/0.25(0.375,S)	2/0.25(0.375,S)
BMU00029	>16	1	0.25	0.5	2	4/0.25(0.375,S)	0.25/0.25(1.008,I)	4/0.25(0.625,I)	0.25/2(1.008,I)
BMU00030	8	0.5	0.25	0.5	2	4/0.125(0.75,I)	0.25/0.25(1.031,I)	1/0.25(0.625,I)	2/0.5(0.5,S)
BMU00031	>16	0.5	0.25	0.5	4	16/0.125(0.75,I)	0.25/0.25(1.008,I)	8/0.125(0.5,S)	0.25/4(1.008,I)
BMU00034	>16	1	0.25	1	4	2/0.25(0.313,S)	4/0.063(0.375,S)	1/0.125(0.156,S)	0.25/4(1.008,I)
BMU00035	16	1	0.125	0.25	4	4/0.25(0.5,S)	4/0.031(0.5/S)	0.5/0.063(0.281,S)	0.25/4(1.008,I)
BMU00036	>16	0.5	0.063	0.25	2	2/0.063(0.188,S)	0.25/0.063(1.008,I)	1/0.063(0.281,S)	2/0.25(0.313,S)
BMU00037	>16	0.5	0.25	0.5	4	8/0.063(0.375,S)	0.25/0.25(1.008,I)	2/0.063(0.186,S)	16/1(0.75,I)
BMU00039	>16	0.5	0.125	0.25	4	16/0.125(0.75,I)	0.25/0.125(1.008,I)	4/0.063(0.375,S)	0.25/4(1.008,I)
BMU00040	>16	0.25	0.063	0.125	2	1/0.0313(0.158,S)	0.25/0.063(1.008,I)	0.5/0.031(0.266,S)	0.5/0.5(0.266,S)
BMU00041	>16	0.5	0.125	0.125	4	4/0.125(0.375,S)	0.25/0.125(1.008,I)	4/0.031(0.383,S)	0.25/4(1.008,I)
ATCC204204	>16	0.5	1	0.5	>4	1/0.125(0.281,S)	1/0.5(0.531,I)	1/0.125(0.281,S)	4/4(0.625,I)
ATCC22019	1	0.125	0.031	0.25	1	0.5/0.031(0.75,I)	0.25/0.031(1.25,I)	0.5/0.031(0.625,I)	0.5/0.25(0.75,I)

a S (synergism, FICI of ≤ 0.5); I (no interaction, 0.5 < FICI ≤ 4); EVL, everolimus; ITC, itraconazole; VRC, voriconazole; POS, posaconazole; AMB, amphotericin B.

### ROS activity.

**FIG 1:**
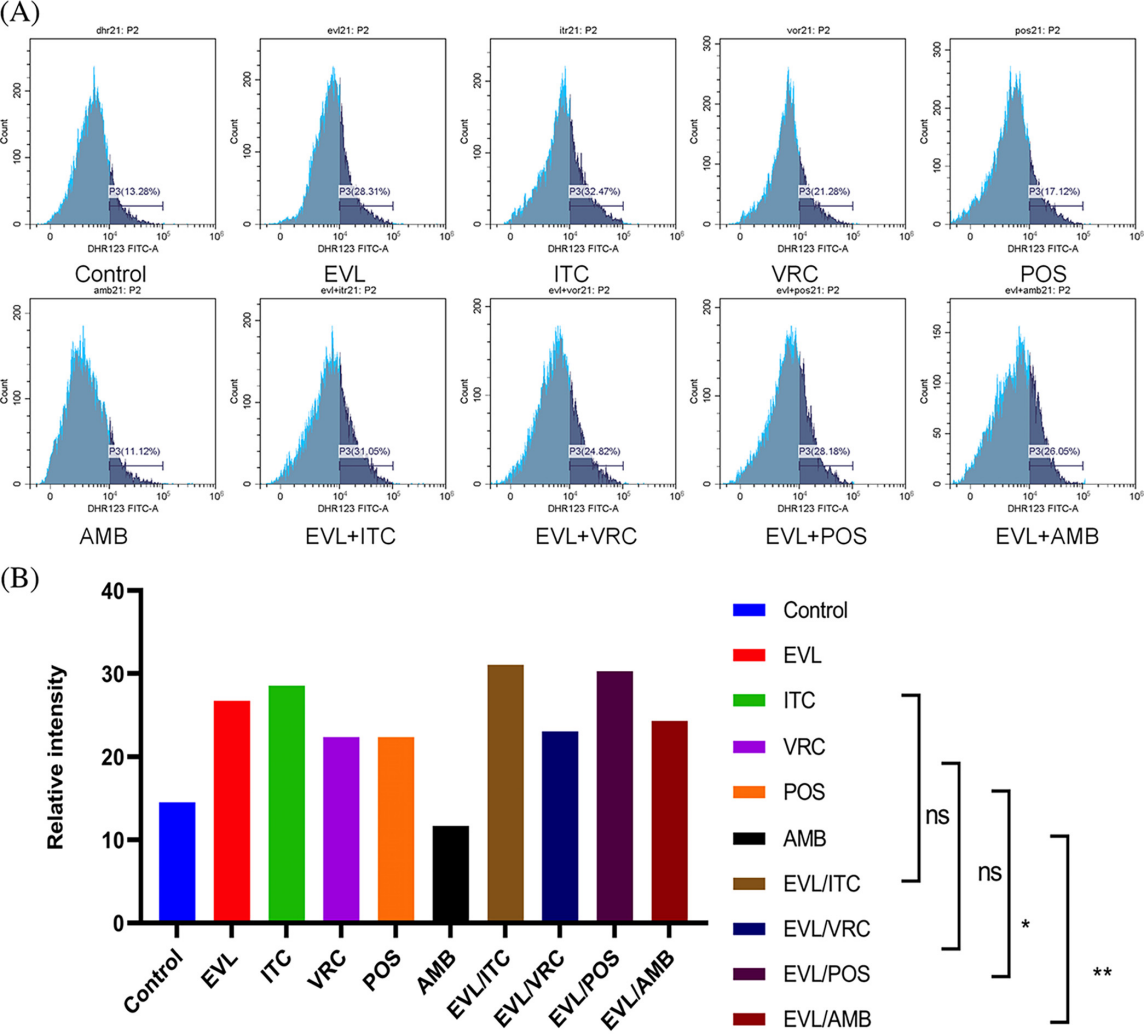
ROS activity level changes in the single and combined groups. The proportion of DHR-123 oxidized by ROS in the control and experimental groups and related statistical analysis. (A) Changes in ROS generation ratio across the single and combined drug groups. In flow cytometry analysis, the abscissa represents the relative fluorescence intensity, and the ordinate represents the spore count. The dark blue area represents the percentage of spores (P3) with emitted fluorescence after DHR-123 was oxidized. The peak value in the dark blue area indicates the largest number of oxidized spores under the corresponding fluorescence intensity. (B) The ROS activity levels were significantly increased in the group treated with EVL plus POS or AMB compared to POS or AMB alone group, respectively. Dihydrorhodamine 123, DHR-123; EVL, everolimus; ITC, itraconazole; VRC, voriconazole; POS, posaconazole; AMB, amphotericin B. *, *P* < 0.05; **, *P* < 0.01; ns, no significance.

When *E. dermatitidis* strain was pretreated with ITC, VRC, POS, or AMB combined with EVL, ROS activity levels were increased by 2.5%, 0.71%, 7.92% (*P* < 0.05), and 12.62% (*P* < 0.05), respectively, compared to ROS activity levels when pretreated with the correspond antifungal agent alone. The results are shown in Fig. 1.

### Use of RT-qPCR to explore the expression levels of antifungal susceptibility-related genes.

**FIG 2:**
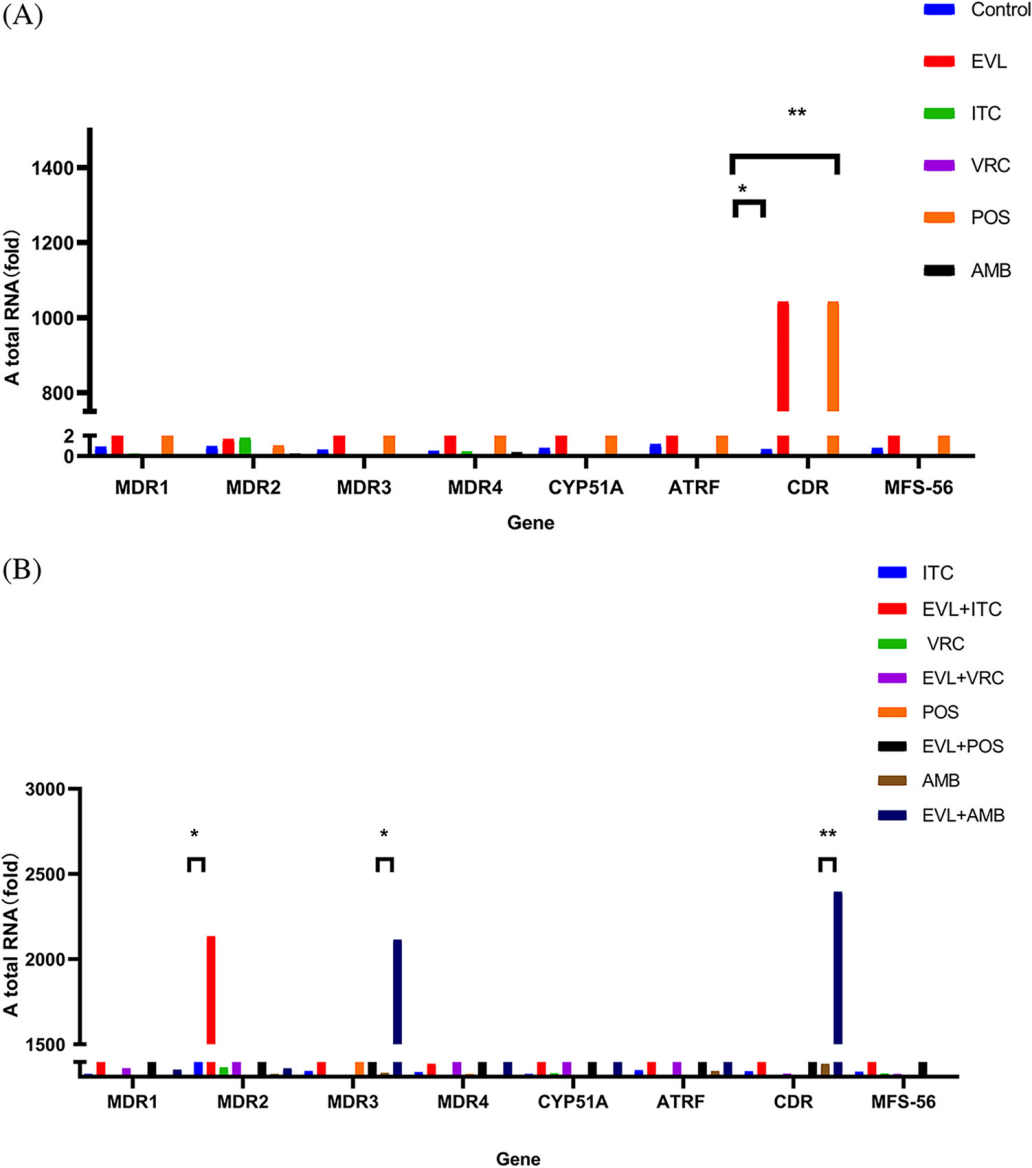
The results of antifungal sensitivity related gene RT-qPCR in the single and combined groups. Note: Total RNA comparison of different genes in drug-free and single-drug groups (A), and total RNA comparison of different genes in single-drug and combination groups (B). EVL, everolimus; ITC, itraconazole; VRC, voriconazole; POS, posaconazole; AMB, amphotericin B. *, *P* < 0.05; **, *P* < 0.015; ns, no significance.

We quantified the expression of *MDR1*, *MDR2*, *MDR3*, *MDR4*, *ATRF*, *CDR1B*, *MFS56*, and *CYP51A*. Compared to antifungal agent mono-treatment, the combination of EVL with ITC resulted in suppression of the expression of the efflux pump *MDR2* (*P* < 0.05), while the expression levels of *MDR1*, *MDR3*, *MDR4*, *ATRF*, *CDR1B*, *MFS56*, and *CYP51A* did not show significant changes (*P > *0.05). Compared to AMB alone, the combination of EVL with AMB suppressed the expression of *MDR3* (*P* < 0.05) and *CDR1B* (*P* < 0.02). However, when EVL was combined with VRC or POS, there was no significant change observed in the expression of antifungal sensitivity-related genes (*P > *0.05). The results are shown in Fig. 2.

### Disk diffusion testing.

**FIG 3:**
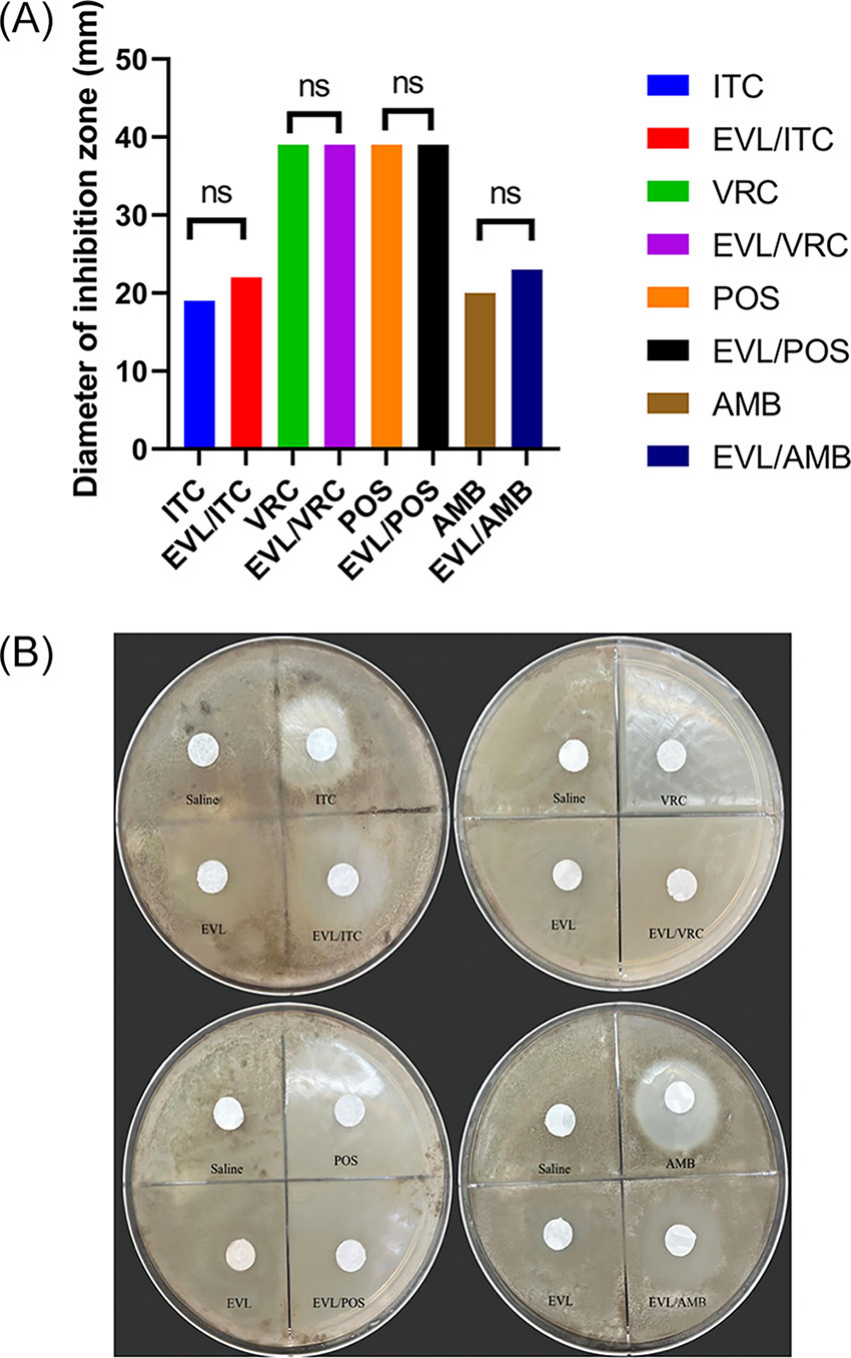
The results of disk diffusion testing of everolimus combined with antifungal agents. Note: Disk diffusion test: *E. dermatitidis* BMU00028 statistical analysis of antifungal agents alone and in combination with everolimus (A). Record the diameter of the inhibition area. The blank tray was impregnated with EVL, ITC, VOR, POS, AMB, EVL + ITC, EVL + VRC, EVL + POS, EVL + AMB, and saline controls (B). EVL, everolimus; ITC, itraconazole; VRC, voriconazole; POS, posaconazole; AMB, amphotericin B; ns, no significance.

In the disk diffusion assay, the paper chip containing EVL did not produce any inhibitory zone for *E. dermatitidis* BMU00028. The combination of everolimus and antifungal drugs (EVL + ITC and EVL + AMB) showed increased diameters in the inhibition zones compared with the single agent (*P > *0.05), but no antagonism was found. The results are shown in Fig. 3 and Table 2.

**TABLE 2:** Drug concentration and diameter of inhibition zone of EVL in combination with antifungal agents^*a*^

Drug	Drug concn (μg)	Diam of inhibition zone (mm)	Drug	Drug concn (μg)	Diam of inhibition zone (mm)
EVL	25.6	0	Saline	-	0
ITC	51.2	19	EVL+ITC	25.6 + 51.2	22
VRC	3.2	39	EVL+VRC	25.6 + 3.2	39
POS	12.8	39	EVL+POS	25.6 + 12.8	39
AMB	19.2	20	EVL+AMB	25.6 + 19.2	22

a EVL, everolimus; ITC, itraconazole; VRC, voriconazole; POS, posaconazole; AMB, amphotericin B. -, not applicable.

### Survival rates increased due to drug combinations.

**FIG 4:**
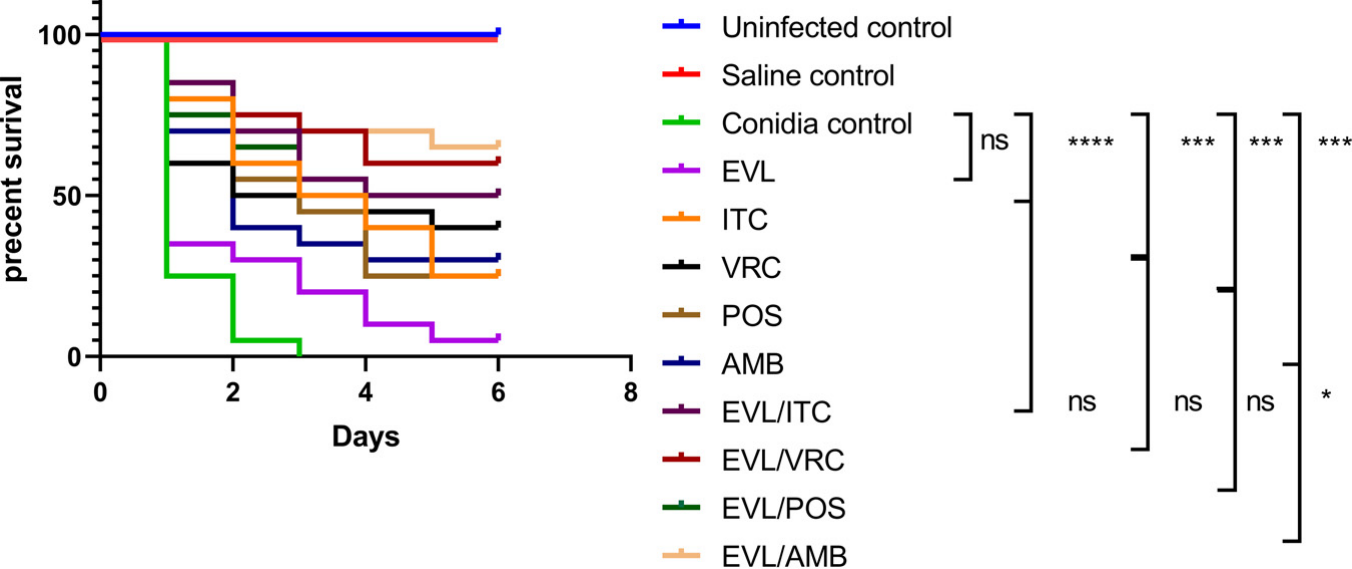
Survival rates in mono-treatment and combination treatment groups. Note: The survival curves of larvae infected with *E. dermatitidis* after different interventions. The control groups of uninfected group and saline group showed all survival. The uninfected and saline controls demonstrated 100% survival at 6 days. Azoles alone and AMB or AMB combine with EVL can significantly improve the survival rate of larvae. EVL, everolimus; ITC, itraconazole; VRC, voriconazole; POS, posaconazole; AMB, amphotericin B; *, *P* < 0.05; ***, *P* ≤ 0.0005; ****, *P* < 0.0001; ns, no significance.

The survival rate was calculated on day 6 in both the control and experimental groups infected with *E. dermatitis.* EVL combined with ITC, VRC, POS, and AMB improved larval survival rates by 25%, 20%, 25%, and 35% (*P* < 0.05), respectively, compared to the mono-treatment of each drug. The results are shown in Fig. 4.

## DISCUSSION

Over the past few years, there has been a significant increase in the occurrence of fungal infections in humans, particularly in immunocompromised patients and those with cancer (11). *E. dermatitidis* is an opportunistic human pathogen found in the environment, mainly in tropical regions. However, it can also survive and grow in artificial habitats such as dishwashers (5, 12–14). It is the most poisonous fungus capable of causing disseminated infections. It can cause severe, deep, and disseminated infections that often recur in humans, and it typically results in fatal outcomes (15). Although antifungal drugs have been shown to be highly effective in killing *E. dermatitis in vitro*, the fungus exhibits low sensitivity to antifungal drugs *in vivo*, resulting in a relatively low cure rate of only 40% to 70% in clinical settings (7, 16–18). As a result, the establishment of drug synergy has become a promising strategy to address the urgent need for novel antifungal methods (17, 18). In recent years, several studies have demonstrated that combining various drugs with azole therapy can result in strong synergistic effects when treating *E. dermatitidis* (18–20), leading to the development of numerous novel therapeutic approaches.

The mTOR pathway, which regulates cell development, proliferation, and survival, is frequently dysregulated in cancer, and EVL, an mTOR inhibitor, is frequently utilized in a variety of malignancies (21). Although there is no rationale for treating fungal infections with EVL, it has been demonstrated that EVL in combination with azoles has a positive synergistic impact (10). Exploring the synergistic effect of EVL and antifungal agents, as well as their putative mechanism of action, can thus serve as a foundation for clinical studies of combination therapy for *E. dermatitidis*. In our *in vitro* experiments, we observed that while EVL alone did not exhibit significant antifungal activity, it did demonstrate strong synergistic effects when used in combination with ITC, POS, and AMB. However, we observed only limited synergy when EVL was combined with VRC. In the disk diffusion experiments, no antagonism was found. Our *in vivo* experiments confirmed that combining EVL with antifungal agents could significantly improve larval survival rates, with the group treated with a combination of EVL and AMB showing the most significant improvement compared to mono-treatment. To further investigate the underlying synergistic mechanisms between antifungal agents and EVL, we conducted ROS activity level testing and quantified the expression of antifungal susceptibility-related genes. ROS is a toxic metabolite produced as a by-product of electron transfer reactions in the respiratory chain, and overproduction of ROS can lead to oxidative stress (22, 23). Our results showed that combining EVL with POS or AMB stimulated ROS activity, which could potentially contribute to the observed synergy effects.

Multiple genes have been implicated in antifungal susceptibility and resistance, including the *CYP51A* gene and genes encoding efflux pumps (24). In this experiment, the expression of *MDR1*, *MDR2*, *MDR3*, *MDR4*, *ATRF*, *CDR1B*, *MFS56*, and *CYP51A* was quantified. When the combination of EVL and ITC was compared to mono-treatment, the expression level of the *MDR2* gene was inhibited significantly (*P < *0.05), while the expression levels of *MDR1*, *MDR3*, *MDR4*, *ATRF*, *CDR1B*, *MFS56*, and *CYP51A* were not significantly altered (*P* > 0.05). Meanwhile, when the combination of EVL and AMB was compared to mono-treatment, the expression level of both *MDR3* and *CDR1B* was decreased (*P* < 0.05). The combination of EVL with VRC or POS did not significantly alter the expression levels of *MDR1*, *MDR2*, *MDR3*, *MDR4*, *ATRF*, *CDR1B*, *MFS56*, and *CYP51A* compared to their respective mono-treatment treatments (*P > *0.05). Aspergillus fumigatus with high expression of *CDR1B* showed resistance to ITC (25). Increased expressions of antifungal sensitivity-related genes, such as *MDR2* and *MDR3*, can cause Botrytis cinerea to exhibit increased efflux activity of fungicides, ultimately leading to resistance (26). The synergistic effects of EVL and AMB or ITC could potentially reduce the expression of resistance genes and increase fungal sensitivity to drugs.

The mTOR signaling pathway has been implicated in drug resistance in cancer (27). EVL, an oral mTOR inhibitor, is widely used as an immunomodulatory agent in the treatment of neuroendocrine tumors, renal cancer, breast cancer, and other diseases (21). Invasive fungi, such as *E. dermatitis*, often cause infections that are more likely to occur in individuals with weakened immune systems, such as those with immunosuppression or cancer patients (28–32). EVL may inhibit various fungal drug pumps that contribute to the synergistic mechanisms of EVL and antifungal drugs against *E. dermatitidis*. Furthermore, the synergistic effect of EVL with azoles or AMB may be attributed to an increase in ROS level activity and the suppression of drug-resistance genes. Based on our study, the combination of EVL with azoles or AMB could be considered a potential treatment option for individuals infected with E. dermatitidis, particularly those who are already using EVL as a primary medication for an underlying disease. This approach offers patients a new therapeutic option and concept. Indeed, studies have demonstrated that there is an interaction between antifungal drugs and EVL, and determining the appropriate dose of everolimus to avoid overexposure is an important consideration. However, despite these challenges, the use of EVL in combination with antifungal agents offers promising new possibilities for the treatment of fungal infections.

## MATERIALS AND METHODS

### Fungal strains, antifungals, and chemical agents.

In this study, 16 clinically isolated strains of *E. dermatitidis* were included (33). All the strains were reconfirmed by performing microscopic morphology and molecular identification techniques. Sequenced DNA (rDNA) was extracted from internal transcribed spacer (ITS) ribosomes to identify the fungi. Susceptibility testing was also performed with C. parapsilosis ATCC22019 and A. flavus ATCC204304 as control strains to ensure quality control. All strains required for this experiment were cultured on Sabouraud Dextrose Agar (SDA, Haibo Biological) at 35°C for 2 to 3 days. Subsequently, susceptibility testing was performed to ensure the viability and purity of the tested fungi. The experimental drugs, such as EVL (number 112991, purity ≥ 99.69%), ITC (number 15455, purity = 99.81%), and AMB (number 62991, purity = 98.0%), were obtained from MedChemExpress (MCE), NJ, USA; VRC (SV8450, purity ≥98%) was obtained from Solarbio, Beijing, China; and POS (S1257, purity ≥ 99.82%) was obtained from Meilunbio, Dalian, China, in powder form. The powder was dissolved in dimethyl sulfoxide to prepare the stock solution (6,400 μg/mL). Working concentrations of azoles and AMB ranged from 0.0313 to 4 μg/mL, while the concentration of EVL ranged from 0.25 to 16 μg/mL. Dihydrorhodamine 123 (DHR-123, No. D1054), a fluorescent dye purchased from Sigma-Aldrich, was mixed into a stock solution (6,400 μg/mL) of dimethyl sulfoxide. Hifair III 1st Strand cDNA Synthesis SuperMix for qPCR (11141ES10) and Hieff UNICON qPCR SYBR green Master MIX (11200ES03) were purchased from Yeasen Biotechnology (Shanghai) Co., Ltd.

### *In vitro* combined drug sensitivity.

According to the previously prescribed procedure, a RPMI 1640 liquid medium (the medium was prepared in accordance with the CLSI microliquid-based dilution method M38-A2, 2008 protocol) was prepared (34). The medium was buffered to pH 7.0 using 3-(N-morpholine) propanesulfonic acid and then sterilized. *In vitro* combined susceptibility testing was performed using the chequerboard method and the antifungal agents were added to 96-well plates utilizing the broth microdilution method. Serial inoculations of 50 μL of diluted antifungal drugs (ITC, VRC, POS, and AMB) were performed vertically. The final concentrations of all four drugs ranged from 0.031 to 4 μg/mL. Serial inoculations of 50 μL of diluted EVL were performed in a horizontal direction with working concentrations ranging from 0.25 to 16 μg/mL. *E. dermatitidis* strains were cultured for 2 to 3 days at 37°C with SDA, and then, the suspended conidia were collected from sterile distilled water. Using a hemocytometer, the concentration was adjusted to 3 to 5 × 10^6^/mL and then finally diluted to a final concentration of 3 to 5 × 10^4^/mL with RPMI 1640. One hundred μL of the 2-fold final concentration of fungal suspension was added. Cultures were then cultivated in an incubator at 35°C for 3 days. The MIC is the concentration that completely inhibits fungal growth, including the MIC values in the single-drug and combination regions for each drug. It is determined by visual identification. All tests for drug susceptibility were repeated thrice. The fractional inhibitory concentration index (FICI) is the MIC value of the combination of two drugs divided by the MIC value of each drug alone. FICI = (MIC A combination)/(MIC A alone) + (MIC B combination)/(MIC B alone), each separately, and the relationship between the interaction of two drugs is represented as follows: FICI ≤ 0.5 synergistic effect, >0.5 and ≤ 4 no interaction, and > 4 antagonism (35).

### ROS activity.

According to the findings of the antifungal test, the combination of EVL and antifungal agents exhibited synergy in the treatment of *E. dermatitidis* BMU00028 (Table 1). To investigate the probable synergistic effects, spores of *E. dermatitidis* BMU00028 were counted using a hemocytometer and dissolved in 10 mL of Sabouraud dextrose broth (SDB) to achieve a final spore concentration of 5 × 10^6^/mL. Depending on the results of antifungal susceptibility tests, solutions of ITC, VRC, POS, AMB, and EVL were prepared at appropriate concentrations. Working concentrations of ITC, VRC, POS, AMB, and EVL are shown in Table 3. The experimental group (EVL and antifungal agent combinations), control group (mono-drug), negative-control group (no drugs), and blank control group (only spores) were established. DHR-123 concentrations (5 μg/mL) and spore concentrations remained constant across all groups. The drug concentrations used in the experiment are shown in Table 3. Culture samples were incubated on a shaker at 37°C, 130 rpm, for 30 min. The spores were then collected, cleaned, and cultivated for another 60 min. The spores were resuspended in 1 mL of PBS. For multiple flow cytometry (B53000, Beckman Kurt, USA) events, one solutions of roughly 150 μL were utilized. A total of 10,000 events were performed and assessed by CytExpert. The test was repeated thrice.

**TABLE 3:** Drug concentrations for ROS activity detection and RT-qPCR^*a*^

Control groups	concn (μg/mL)	Exptl groups	Concn (μg/mL)
EVL	16	EVL/ITC	2/0.25
ITC	1	EVL/VOTR	0.25/0.0625
VRC	0.0625	EVL/POS	2/0.25
POS	1	EVL/AMB	2/0.25
AMB	1		

a EVL, everolimus; ITC, itraconazole; VRC, voriconazole; POS, posaconzaole; AMB, amphotericin B.

### RT-qPCR experiments.

The antifungal susceptibility-related genes (11), including efflux pump genes, such as *MDR1* (GenBank ID: 20312659), *MDR2* (GenBank ID: 20310569), *MDR3* (GenBank ID: 20307294), *MDR4* (GenBank ID: 20306074), *ATRF* (GenBank ID: 20306320), *CDR1B* (GenBank ID: 20306319), and *MFS56* (GenBank ID: 20305787), and the lanosterol 14α-demethylase gene (*CYP51A* [GenBank ID: 20304724]), were quantified to analyze the synergistic effects of EVL and antifungal agents. A final concentration of 1 to 3 × 10^7^/mL of *E. dermatitidis* BMU00028 was dissolved in 10 mL of SDB. A total of 10 groups were included, and based on the results of antifungal susceptibility testing, appropriate concentrations of azoles, AMB, and EVL solutions were prepared. The working concentrations of azoles, AMB, and EVL solutions were consistent with those used in the experiments described above (Table 3). No drug group served as the blank control, and EVL, ITC, VRC, POS, and AMB were added to the control group. In the experimental group, ITC + EVL, VRC + EVL, POS + EVL, and AMB + EVL were added sequentially and shaken at 130 rpm and 37°C for 4 h in a shaker box. The cultured fungal solution was centrifuged (2,000 to 4,000 rpm) for 10 min. After taking out the supernatant, 5 mL of precooled PBS was used to wash it twice, and finally, solid tissues were centrifuged at 4,000 rpm for 5 min. RNA was extracted from drug-stimulated fungal tissues using Total RNA Extraction Reagent and chloroform, and the process is briefly described as follows: Total RNA Extraction Reagent (1 mL) was added to a 1.5 mL EP tube containing 50 to 100 mg of stimulated fungal tissues. The RNA was extracted using TRIzol solution, and the total RNA was separated from the DNA and protein, followed by centrifugation. Next, chloroform was added and the total RNA was retained in the upper aqueous phase. The total RNA was then recovered by precipitation with isopropanol. The RNA was obtained by adding 50 μL of sterile water. After performing reverse transcription using Hifair III 1st Strand cDNA Synthesis SuperMix, the obtained cDNA was immediately used for qPCR with Hieff UNICON qPCR SYBR green Master MIX. This experiment was repeated thrice. A list of RT-qPCR primers is provided in Table 4.

**TABLE 4:** Primers of RT-qPCR-related genes

Name	Sequence (5′–3′)	Function
actin-F	5′-CTGTGCACATTGTCGCCAGGG-3′	Amplify the actin (GenBank ID:20309310)
actin-R	5′-GTCCAGATTAAGCTGTCGCGC-3′	
MDR1-F	5′-GTCCTGCTGCCGTTTCGT-3′	Amplify the MDR1 (GenBank ID:20312659)
MDR1-R	5′-TCGCATTTGTCGGGTTGA-3′	
MDR2-F	5′-AGAGGGTACAAACACTATGGC-3′	Amplify the MDR2 (GenBank ID:20310569)
MDR2-R	5′-CTGAGGTGGTGAAGAAAGAT-3′	
MDR3-F	5′-CCATCGCAGCAATCGTAA-3′	Amplify the MDR3 (GenBank ID:20307294)
MDR3-R	5′-TGCCCAGTAAGTCTGTTTCA-3′	
MDR4-F	5′-TCGCAGCAATCGTAACCG-3′	Amplify the MDR4 (GenBank ID:20306074)
MDR4-R	5′-TCGTGCCCACCAGAAAGG-3′	
CYP51A-F	5′-ACCTGTATTTGGCGAAGG-3′	Amplify the CYP51A (GenBank ID:20304724)
CYP51A-R	5′-GAGATCAGATCCACGTATGTTT-3′	
ATRF-F	5′-CCTGACCTATACCGACTCGTC-3′	Amplify the ATRF (GenBank ID:20306320)
ATRF-R	5′-GCTGTCTCTTTGATTGGCGA-3′	
CDR1B-F	5′-GTGCTCAATTGCTCAAAGACATTG-3′	Amplify the CDR1B (GenBank ID:20306319)
CDR1B-R	5′-GGAGATTGTAGAAGATGGAACCG-3′	
MFS56-F	5′-CGCGTCCCACTTCAACAAATTT-3′	Amplify the MFS56 (GenBank ID: 20305787)
MFS56-R	5′-TCATTAGCTGGAAGATACTGGTGT-3′	

### Disk diffusion testing.

*E. dermatitidis* BMU00028 were cultured on an SDA medium according to standard protocols. The culture was incubated at 35°C for 2 days, after which colonies were carefully picked using sterile cotton swabs and transferred to a 1.5 mL EP tube containing sterilized water. The prepared suspensions were spread evenly onto RPMI 1640 agar plates and left to dry, and the conidia concentrations were adjusted to between 1 × 10^6^ and 5 × 10^6^ CFU/mL using a hemocytometer for counting. Circular paper sheets with a diameter of 8 mm were spaced equally on the agar plate. Different concentrations of one or more drugs were dropped onto the center of the paper sheet. Saline was used as the control group, while a single agent was used as another control group. The experimental group consisted of a combination of drugs. The dried sheets were incubated at 35°C for 48 to 72 h. Following incubation, the diameter of the inhibition zone was measured. This experiment was repeated thrice.

### *In vivo* model for antifungal tests.

To evaluate the *in vivo* effects of EVL alone and in combination with antifungal drugs, sixth instar larvae of G. mellonella, weighing approximately 300 mg and sourced from Sichuan, China, were infected with *E. dermatitidis* BMU00028. Twelve experimental groups were created, each consisting of 20 G. mellonella. The control groups included an uninfected group, a sterile saline group, and a group infected with a conidial suspension. To the experimental groups, EVL, ITC, VRC, POS, AMB, EVL+ITC, EVL + VRC, EVL + POS, and EVL + AMB were added. The appropriate drug concentrations were added in accordance with the results obtained from the *in vitro* experiments (Table 3). The larvae used in the experiment were grown in a box in the dark at 37°C. The concentration of cultured BMU00028 was adjusted to 1 × 10^7^ spores/mL using sterile water. Separate injections of 10 mL sterile saline and conidial solution were administered to the groups. No medical treatment was provided to the uninfected group. The injection site was cleaned using an alcoholic cotton swab. Then, using a Hamilton syringe (25-gauge, 50 mL), a conidial suspension was injected into the last gastropod on the left side of the larvae, while drugs were injected into the body. In a box at 37°C in the dark, the larvae were cultured. The survival of the larvae was monitored and recorded for 6 consecutive days at the same time each day. The test was repeated thrice.

### Statistical analysis.

Using CytExpert and GraphPad Prism 8 software, all experimental results were analyzed. Data were shown as mean ± SEM. The Shapiro–Wilks test was used to determine if the ROS activity data and RT-qPCR experiment data had a normal distribution (*P* < 0.05). The group differences were examined using paired *t* test analysis in ROS activity experiments. Intergroup data were analyzed with one-way ANOVA (nonparametric or mixed) in RT-qPCR experiments. The *in vivo* experiments were analyzed using the Mantel–COX test.

### Data availability.

The original contributions presented in the study are included in the article; further inquiries can be directed to the corresponding authors.

## References

[1] Kirchhoff L, Olsowski M, Rath PM, Steinmann J. 2019. Exophiala dermatitidis: key issues of an opportunistic fungal pathogen. Virulence 10:984–998. doi:10.1080/21505594.2019.1596504. 30887863 PMC8647849

[2] Poyntner C, Mirastschijski U, Sterflinger K, Tafer H. 2018. Transcriptome study of an Exophiala dermatitidis PKS1 mutant on an ex vivo skin model: is melanin important for infection? Front Microbiol 9:1457. doi:10.3389/fmicb.2018.01457. 30018609 PMC6037837

[3] Poyntner C, Blasi B, Arcalis E, Mirastschijski U, Sterflinger K, Tafer H. 2016. Exophiala dermatitidis: the transcriptome of during skin model infection. Frontiers in Cellular Infection Microbiology 6:136. doi:10.3389/fcimb.2016.00136. 27822460 PMC5075926

[4] Byrne DD, Reboli AC. 2017. Rare yeast infections: risk factors, clinical manifestations, treatment, and special considerations. Curr Clin Micro Rpt 4:218–231. doi:10.1007/s40588-017-0073-7.

[5] Usuda D, Higashikawa T, Hotchi Y, Usami K, Shimozawa S, Tokunaga S, Osugi I, Katou R, Ito S, Yoshizawa T, Asako S, Mishima K, Kondo A, Mizuno K, Takami H, Komatsu T, Oba J, Nomura T, Sugita M. 2021. Exophiala dermatitidis. World J Clin Cases 9:7963–7972. doi:10.12998/wjcc.v9.i27.7963. 34621853 PMC8462220

[6] Wang C, Xing H, Jiang X, Zeng J, Liu Z, Chen J, Wu Y. 2019. Cerebral phaeohyphomycosis caused by Exophiala dermatitidis in a Chinese CARD9-deficient patient: a case report and literature review. Front Neurol 10:938. doi:10.3389/fneur.2019.00938. 31551907 PMC6734004

[7] Patel A, Patel K, Darji P, Singh R, Shivaprakash M, Chakrabarti A. 2013. Exophiala dermatitidis endocarditis on native aortic valve in a postrenal transplant patient and review of literature on E. dermatitidis infections. Mycoses 56:365–372. doi:10.1111/myc.12009. 23013169

[8] Crespo J, Hall M. 2002. Elucidating TOR signaling and rapamycin action: lessons from Saccharomyces cerevisiae. Microbiol Mol Biol Rev 66:579–591. doi:10.1128/MMBR.66.4.579-591.2002. 12456783 PMC134654

[9] Madeira J, Masuda C, Maya-Monteiro C, Matos G, Montero-Lomelí M, Bozaquel-Morais B. 2015. TORC1 inhibition induces lipid droplet replenishment in yeast. Mol Cell Biol 35:737–746. doi:10.1128/MCB.01314-14. 25512609 PMC4301715

[10] Wang Z, Liu M, Liu L, Li L, Tan L, Sun Y. 2022. The synergistic effect of tacrolimus (FK506) or everolimus and azoles against *Scedosporium* and *Lomentospora* species *in vivo* and *in vitro*. Cell Infect Microbiol 12:864912. doi:10.3389/fcimb.2022.864912. PMC9046971 35493742

[11] Benaducci T, Matsumoto M, Sardi J, Fusco-Almeida A, Mendes-Giannini M. 2015. A flow cytometry method for testing the susceptibility of Cryptococcus spp. to amphotericin B. Rev Iberoam Micol 32:159–163. doi:10.1016/j.riam.2014.06.004. 25639695

[12] Poyntner C, Mirastschijski U, Sterflinger K, Tafer H. 2018. Exophiala dermatitidis PKS1 transcriptome study of an mutant on an skin model: is melanin important for Infection? 9:1457. doi:10.3389/fmicb.2018.01457. PMC6037837 30018609

[13] Jayaram M, Nagao H. 2020. First report of environmental isolation of Exophiala spp. in Malaysia. Curr Microbiol 77:2915–2924. doi:10.1007/s00284-020-02109-w. 32661678

[14] Zupančič J, Novak Babič M, Zalar P, Gunde-Cimerman N. 2016. The Black Yeast Exophiala dermatitidis and other selected opportunistic human fungal pathogens spread from dishwashers to kitchens. PLoS One 11:e0148166. doi:10.1371/journal.pone.0148166. 26867131 PMC4750988

[15] Song Y, da Silva N, Weiss V, Vu D, Moreno L, Vicente V, Li R, de Hoog G. 2020. Exophiala dermatitidis: comparative genomic analysis of capsule-producing black yeasts and, potential agents of disseminated mycoses. Front Microbiol 11:586. doi:10.3389/fmicb.2020.00586. 32373085 PMC7179667

[16] Kondori N, Gilljam M, Lindblad A, Jönsson B, Moore E, Wennerås C. 2011. High rate of Exophiala dermatitidis recovery in the airways of patients with cystic fibrosis is associated with pancreatic insufficiency. J Clin Microbiol 49:1004–1009. doi:10.1128/JCM.01899-10. 21209163 PMC3067733

[17] Revankar S, Sutton D. 2010. Melanized fungi in human disease. Clin Microbiol Rev 23:884–928. doi:10.1128/CMR.00019-10. 20930077 PMC2952981

[18] Gao L, Sun Y, He C, Zeng T, Li M. 2018. Synergy between pyrvinium pamoate and azoles against Exophiala dermatitidis. Antimicrobial Agents Chemotherapy 62:e02361-17. doi:10.1128/aac.02361-17. 29437619 PMC5913963

[19] Gao L, Sun Y, He C, Li M, Zeng T. 2018. In vitro interactions between 17-AAG and azoles against Exophiala dermatitidis. Mycoses 61:853–856. doi:10.1111/myc.12824. 29998564

[20] Sun Y, Gao L, He C, Li M, Zeng T. 2018. In vitro interactions between IAP antagonist AT406 and azoles against planktonic cells and biofilms of pathogenic fungi Candida albicans and Exophiala dermatitidis. Med Mycol 56:1045–1049. doi:10.1093/mmy/myx150. 29346584

[21] Hasskarl J. 2018. Everolimus. Recent Results Cancer Res 211:101–123. doi:10.1007/978-3-319-91442-8_8. 30069763

[22] Majumder D, Nath P, Debnath R, Maiti D. 2021. Understanding the complicated relationship between antioxidants and carcinogenesis. J Biochem Mol Toxicol 35:e22643. doi:10.1002/jbt.22643. 32996240

[23] Marschall R, Tudzynski P. 2016. Reactive oxygen species in development and infection processes. Semin Cell Dev Biol 57:138–146. doi:10.1016/j.semcdb.2016.03.020. 27039026

[24] Pérez-Cantero A, López-Fernández L, Guarro J, Capilla J. 2020. Azole resistance mechanisms in Aspergillus: update and recent advances. Int J Antimicrob Agents 55:105807. doi:10.1016/j.ijantimicag.2019.09.011. 31542320

[25] Fraczek M, Bromley M, Buied A, Moore C, Rajendran R, Rautemaa R, Ramage G, Denning D, Bowyer P. 2013. The cdr1B efflux transporter is associated with non-cyp51a-mediated itraconazole resistance in Aspergillus fumigatus. J Antimicrob Chemother 68:1486–1496. doi:10.1093/jac/dkt075. 23580559

[26] Kretschmer M, Leroch M, Mosbach A, Walker AS, Fillinger S, Mernke D, Schoonbeek HJ, Pradier JM, Leroux P, De Waard MA, Hahn M. 2009. Fungicide-driven evolution and molecular basis of multidrug resistance in field populations of the grey mould fungus Botrytis cinerea. PLoS Pathog 5:e1000696. doi:10.1371/journal.ppat.1000696. 20019793 PMC2785876

[27] Akbarzadeh M, Mihanfar A, Akbarzadeh S, Yousefi B, Majidinia M. 2021. Crosstalk between miRNA and PI3K/AKT/mTOR signaling pathway in cancer. Life Sci 285:119984. doi:10.1016/j.lfs.2021.119984. 34592229

[28] Hoenigl M, Salmanton-Garcia J, Walsh TJ, Nucci M, Neoh CF, Jenks JD, Lackner M, Sprute R, Al-Hatmi AMS, Bassetti M, Carlesse F, Freiberger T, Koehler P, Lehrnbecher T, Kumar A, Prattes J, Richardson M, Revankar S, Slavin MA, Stemler J, Spiess B, Taj-Aldeen SJ, Warris A, Woo PCY, Young JH, Albus K, Arenz D, Arsic-Arsenijevic V, Bouchara JP, Chinniah TR, Chowdhary A, de Hoog GS, Dimopoulos G, Duarte RF, Hamal P, Meis JF, Mfinanga S, Queiroz-Telles F, Patterson TF, Rahav G, Rogers TR, Rotstein C, Wahyuningsih R, Seidel D, Cornely OA. 2021. Global guideline for the diagnosis and management of rare mould infections: an initiative of the European Confederation of Medical Mycology in cooperation with the International Society for Human and Animal Mycology and the American Society for Microbiology. Lancet Infect Dis 21:e246–e257. doi:10.1016/S1473-3099(20)30784-2. 33606997

[29] Itoh N, Murakami H, Ishibana Y, Matsubara Y, Yaguchi T, Kamei K. 2021. Challenges in the diagnosis and management of central line-associated blood stream infection due to Exophiala dermatitidis in an adult cancer patient. J Infect Chemother 27:1360–1364. doi:10.1016/j.jiac.2021.04.009. 33888421

[30] Maraki S, Katzilakis N, Neonakis I, Stafylaki D, Meletiadis J, Hamilos G, Stiakaki E. 2022. Exophiala dermatitidis central line-associated bloodstream infection in a child with Ewing's sarcoma: case report and literature review on paediatric infections. Mycopathologia 187:595–602. doi:10.1007/s11046-022-00658-1. 35994217

[31] Nakatani R, Ashiarai M, Yoshihara H, Yada K, Nozaki T, Ushigusa T, Mori N, Hasegawa D. 2022. Multidisciplinary management of disseminated Exophiala dermatitidis mycosis in an infant with mixed phenotype acute leukemia: a case report. BMC Infect Dis 22:797. doi:10.1186/s12879-022-07773-w. 36274136 PMC9590134

[32] Chalkias S, Alonso CD, Levine JD, Wong MT. 2014. Emerging pathogen in immunocompromised hosts: Exophiala dermatitidis mycosis in graft-versus-host disease. Transpl Infect Dis 16:616–620. doi:10.1111/tid.12236. 24890324

[33] Sun Y, Tan L, Yao Z, Gao L, Yang J, Zeng T. 2022. In vitro and interactions of TOR inhibitor AZD8055 and azoles against pathogenic fungi. Microbiol Spectr 10:e0200721. doi:10.1128/spectrum.02007-21. 35019705 PMC8754115

[34] John HR, Barbara DA, David A, Beth AS, Steven DB, Vishnu C. 2008. Reference method for broth dilution antifungal susceptibility testing of filamentous fungi, approved standard. Second edition. M38-A2. National Committee for Clinical Laboratory Standards.

[35] Odds F. 2003. Synergy, antagonism, and what the chequerboard puts between them. J Antimicrob Chemother 52:1. doi:10.1093/jac/dkg301. 12805255

